# The Identification of Cotton Fibers Dyed with Reactive Dyes for Forensic Purposes

**DOI:** 10.3390/molecules25225435

**Published:** 2020-11-20

**Authors:** Daria Śmigiel-Kamińska, Jolanta Wąs-Gubała, Piotr Stepnowski, Jolanta Kumirska

**Affiliations:** 1 Faculty of Chemistry, University of Gdańsk, ul. Wita Stwosza 63, 80-308 Gdansk, Poland; d.smigiel-kaminska@phdstud.ug.edu.pl (D.Ś.-K.); piotr.stepnowski@ug.edu.pl (P.S.); 2 Institute of Forensic Research, Microtrace Analysis Section, Westerplatte 9, 31-033 Krakow, Poland; jwas@ies.krakow.pl

**Keywords:** cotton fibers, dyed fibers, reactive dyes, forensic analysis, chromatographic methods, spectroscopic methods

## Abstract

Some of the most common microtraces that are currently collected at crime scenes are fragments of single fibers. The perpetrator leaves them at a crime scene or takes them away, for example, on their clothing or body. In turn, the microscopic dimensions of such traces mean that the perpetrator does not notice them and therefore usually does not take action to remove them. Cotton and polyester fibers dyed by reactive and dispersion dyes, respectively, are very popular within clothing products, and they are hidden among microtraces at the scene of a crime. In our recently published review paper, we summarized the possibilities for the identification of disperse dyes of polyester fibers for forensic purposes. In this review, we are concerned with cotton fibers dyed with reactive dyes. Cotton fibers are natural ones that cannot easily be distinguished on the basis of morphological features. Consequently, their color and consequently the dye composition are often their only characteristics. The presented methods for the identification of reactive dyes could be very interesting not only for forensic laboratories, but also for scientists working in food, cosmetics or pharmaceutical/medical sciences.

## 1. Introduction

The experts from forensic laboratories are able to establish relationships between people, objects and a crime scene on the basis of the study of microtraces, very often in the form of fragments of single fibers, which sometimes also help to reconstruct the circumstances of an event. This means that even the small fragment of a fiber can be used as evidence in legal proceedings regarding such events as homicides, sexual crimes, assaults, or road accidents. The main purpose of identifying and comparing single fibers is to determine their physicochemical features and then classify them and determine if they originate from a known source. In the case of fibers belonging to the same type, e.g., cotton fibers, color plays an important role in their comparative study. Colors may be specified by 15 different color formats such as RGB, CMYK, HSV, HSL, CIELab, Android, Decimal, and YUV. For example, as an RGB triplet, a color is specified according to the intensity of its red, green, and blue components, each represented by eight bits. Thus, there are 24 bits used to specify a web color within the sRGB gamut, and 16,777,216 colors that may be so specified. The RYB color format or color space builds the colors from a combination of red, yellow, and blue. Each of these color values defines the intensity of the color as an integer value from 0 to 255. The value 255 stands for 100% intensity for the color value, so an RYB color of 0, 255, 0 would describe yellow.

In the forensic laboratory, color is established firstly with the use of optical microscopy techniques, and then, on this basis, the evidential fibers are qualified for further research using more advanced analytical methods. So far, it has mainly concerned UV-Vis microspectrophotometry [1], Raman spectroscopy [2], and more traditional and low-cost chromatographic methods, such as thin-layer chromatography (TLC) [3]. Single cotton fibers comprise the most common group of microtraces, and these fibers have been part of the human environment since ancient times. This is confirmed by population studies conducted so far on extraneous fibers present on different surfaces [4] and, for example, studies showing that such fibers accumulate even in regions as remote as the Arctic [5].

Reactive dyes, including a number of their improved structures and mixtures, are used to dye these particularly widespread fibers. There are a limited number of chromatographic methods settled for the comparison of cotton fibers dyed with reactive dyes, and no review paper has been published concerning this subject more broadly.

The analysis of the textile market shows that cotton dyed with reactive dyes and polyester dyed with disperse dyes are the most popular fibers within clothing products. In our recently published review paper [6], we summarized the possibilities for the identification of polyester fibers dyed with disperse dyes for forensic purposes. In this review, we are concerned with cotton fibers dyed with reactive dyes. Therefore, apart from the specificity of reactive dyes used for dyeing cotton in the textile industry, we intend to discuss the possibilities of using specific research methods in the analysis of this type of materials, i.e., chromatographic and spectroscopic ones.

## 2. Literature Review Sections

### 2.1. Reactive Dyes in the Textile Industry—An Overview

Textile dyeing of fibers, yarns, or fabrics is the aqueous application of color, most commonly using synthetic organic dyes. Dye and auxiliary processing chemicals are incorporated into the textiles in this process so as to achieve a uniform depth of color and color fastness properties suitable for the end use. For the latter, various types of dyes and chemical additives as well as various technological processes are used [7,8,9,10,11].

When dyeing on wool, silk, cotton, and regenerated cellulosic fibers, reactive dyes offer excellent fastness properties. They are also a very effective class of modern synthetic dyes due to the offered range of shades and flexibility in use. Reactive dyes may be broadly defined as chromophores containing pendant groups that are capable of forming covalent bonds with nucleophilic sites in fibrous substrates. If these bonds remain stable under the conditions of laundering, the final user can expect excellent the wash fastness properties of the coloration. Lewis gave detailed information on the development of both the chemistry of reactive systems and the chemical technology related to the application of reactive dyes to different types of fibers [12].

**Figure 1:**
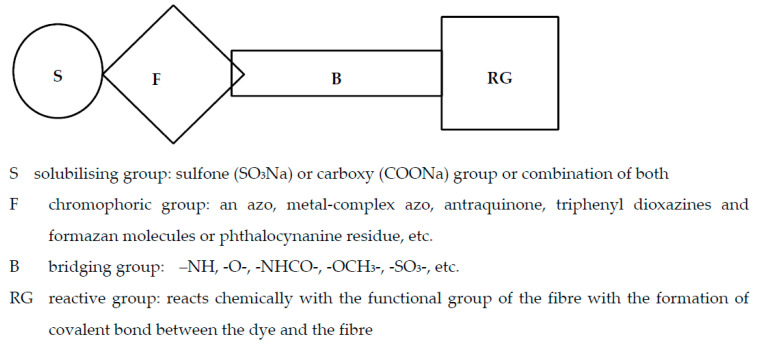
The general formula of reactive dyes.

**Figure 2:**
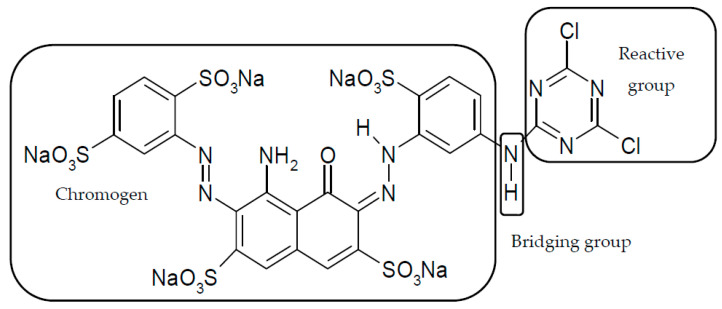
Molecular structure of a reactive dye—C. I. Reactive Blue 109. Reprinted from Parimal P., Industrial Water Treatment Process Technology, Pages 243–511, Copyright (2017), with permission from Elsevier [22].

Reactive dyes have many parts: a color part, a solubilizing part, and a reactive part [13,14,15,16,17,18,19,20,21]. They form covalent bonds with the hydroxyl groups of the fibers, as a result of which the dyes are characterized by a good resistance to wet factors [13,14,15,16,17,18,19,20,21]. Compounds used to develop a bond between a textile fiber and a dye are generally called mordants. The structure of reactive dyes can be presented as a diagram, as shown in Figure 1, and an example of reactive dyes is shown in Figure 2 [22].

This class of dyes has become increasingly popular since the 1950s, when the first commercial reactive dyes for cotton were introduced, and to date, a number of improved dye structures and mixtures have been introduced into practice. However, the use of these dyes results in disagreeable levels of dissolved solids and oxygen demand in effluent, and the remaining unfixed dye on cotton and the hydrolyzed dyes also contribute to this pollution. Khatri et al. [23] reviewed the options to progress the sustainability of the dyeing process through the improvement of dyeing machinery and processes, the chemical modification of cotton fiber prior to dyeing, the development of reactive dyes, the use of biodegradable organic compounds in dyebath formulations, and effluent treatment processes. The use of two reactive groups in a dye molecule, resulting in higher fixation efficiencies (bi-functional reactive dyes), and incorporating more than two reactive groups into the dye molecule, which should theoretically further increase the fixation efficiency (polyfunctional dyes), have been indicated as example advances for reducing effluent pollution. For example, other options are reactive dyes fixable at neutral pH, acid-fixing reactive dyes, low-salt reactive dyes, and cationic reactive dyes [23].

Reactive dyes can be characterized by the following five main types [15].

1. MCT/VS bi-functional dyes—these are reactive dyes containing a monochlorotriazinyl group and a vinyl sulfone group, and they are useful for the dyeing of cellulose fibers.
2. VS dyes—these are reactive dyes containing a vinyl sulfone group, and they are useful for the dyeing of cellulose fibers.
3. MCT/MCT bi-functional dyes—these are reactive dyes containing two monochlorotriazinyl groups and characterized by high fixation in dyeing polyester/cotton blends.
4. MCT dyes—these are reactive dyes containing a monochlorotriazinyl group.
5. DCT dyes—these are reactive dyes containing a dichloro triazinyl group.

Reactive dyes cover the full color palette with vivid shades. Therefore, the following color groups can be presented: yellows, oranges, reds, purples, navy blues and blacks, blues. Reactive yellows are azo dyes such as aromatic amines, pyrazolates, and pyridones and their derivatives. The chromophore system of the dye molecule consists of one or two isolated azo bonds. Reactive oranges are azo dyes that are mainly derivatives of 6-amino-1-hydroxynaphtalene-3-sulfonic acid. Reactive reds are dyes such as N-acylated H acids (1-amino-8-hydroxynaphtalene-3,6-disulfonic acid) and often contain one or two isolated azo bonds. The next reactive dyes are purples. These dyes are mainly copper complexes of N-acylated H acid (o,o′-hydroxyazo). Reactive dyes such as navy blues and blacks are diazo compounds. These dyes are obtained by double coupling with H acid [13].

Table 1 shows the characteristic groups of reactive dyes.

Reactive dyes can be classified in several ways. The first is based on the types of the reactive group: (a) halogen (triazine group, pyridimine group), (b) activated vinyl compound (vinyl sulfone, vinyl acrylamide, vinyl sulfoamide) [13,24]. The next classification is based on the reactivity of the dyes: (a) lower reactive dye/medium reactive dye, (b) higher reactive dye. They can also be classified based on a parameter such as the dyeing temperature: (a) cold brand, (b) medium brand, (c) hot brand [15].

Reactive dyes are the youngest and the most important dye class for cellulosic material. They offer a wide range of dyes of many colors [15].

#### The Formation of a Reactive Dye–Fiber Bond

The necessary condition for a chemical reaction to occur is the physical contact of reactants. The process of the application of reactive dyes on cotton fibers consists of two steps. The first step is the sorption of dyes onto cellulose fibers from the dyeing bath. Reactive dyes contain anionic sulfone groups. These groups make it difficult for dye molecules to enter into physical contact with negatively ionized cellulose fiber. In order to overcome the electrostatic barrier of dye–fiber, the addition of electrolytes is necessary. The most commonly used electrolytes are sodium chloride or disodium sulfate (VI). The process of the sorption of the dye from the bath onto the fiber is carried out in a nearly neutral environment. In the process, dyeing is established between the dye concentration in solution and the fiber.

**Figure 3:**
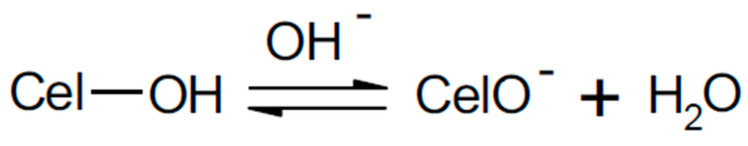
Formation of cellulosate anione. Reprinted by permission from the Springer Nature, Fibers and Polymers, The effect of biodegradable organic acids on the improvement of cotton ink-jet printing and antibacterial activity, Soleimani-Gorgani, A., Karami, Z. Copyright (2017) [25].

**Figure 4:**
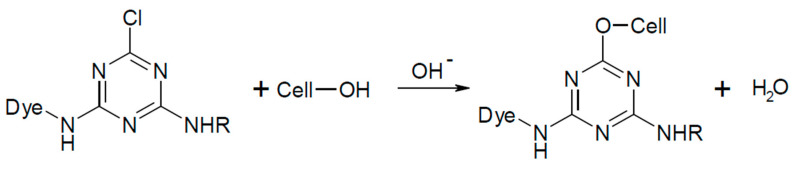
Reaction of a reactive dye with cellulose. Reprinted by permission from the Springer Nature, Fibers and Polymers, The effect of biodegradable organic acids on the improvement of cotton ink-jet printing and antibacterial activity, Soleimani-Gorgani, A., Karami, Z. Copyright (2017) [25].

The second step in the dyeing process is the reaction with the cellulose fiber. The reaction proceeds in the presence of an alkali due to the dye–fiber reaction mechanism. Dye, containing a chlorine of a fluorine atom (triazine, pyrimidine, quinoxaline) in the molecule, reacts with cellulose according to the nucleophilic aromatic substitution mechanism of S_N_2 [13,18,20]. The reaction mechanism of a reactive dye with cellulose is shown in Figure 3 [25] and in Figure 4 [25].

**Figure 5:**
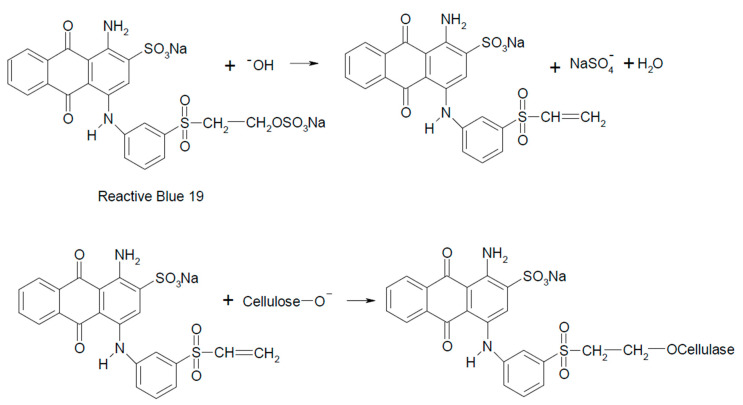
Reaction of Reactive Blue 19 with cellulose.

Dyes containing a vinylsulfonyl moiety as a reactive system or a 2-sulfate ethylsulfonyl moiety, which is a precursor of the vinylsulfonyl moiety, react with cellulose fiber according to the reaction mechanism of nucleophilic attachment to an unsaturated bond [13,18,20]. The example of such a reaction is shown in Figure 5.

### 2.2. Extraction of Reactive Dyes from Dyed Textile Fibers for Forensic Purposes

#### 2.2.1. Cleavage of Reactive Dyes from Dyed Cotton Textiles

**Figure 6:**
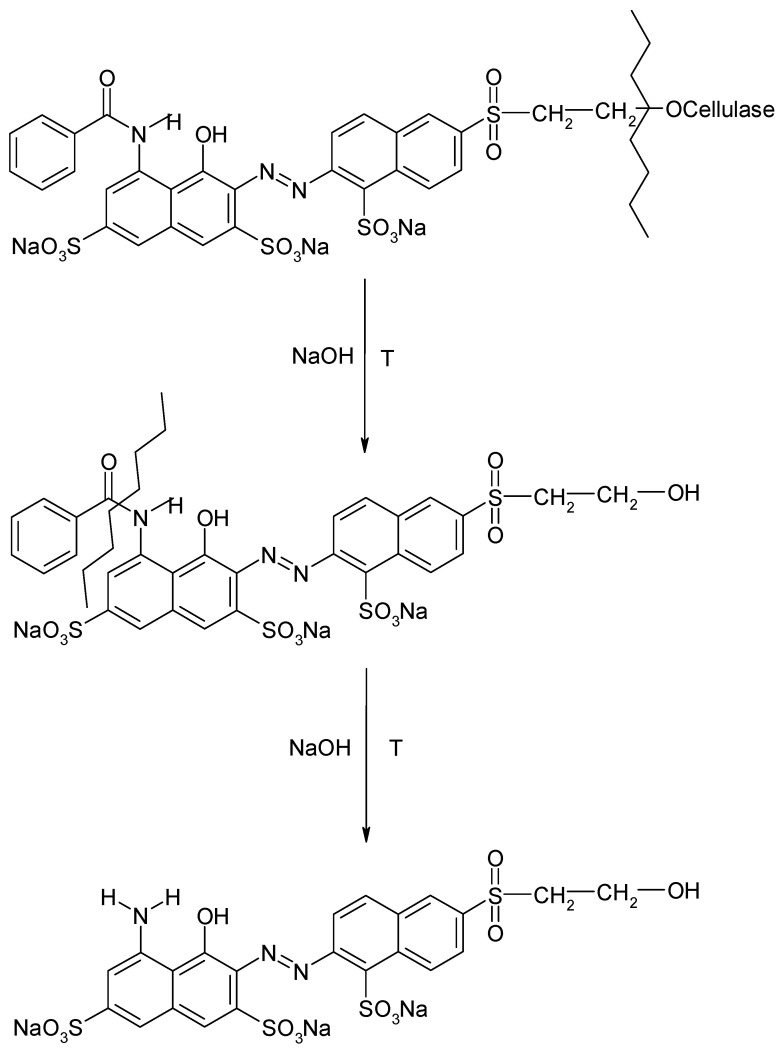
Mechanism of the extraction of Reactive Red 180 from cellulose, involving dye hydrolysis due to excess NaOH.

The conducted studies on the population of fibers have shown that cotton is the most common textile fiber on internal and external surfaces [26,27,28]. Untypical to other textiles, cotton fibers can be dyed using three types of dyes: direct, vat, and reactive. Among them, reactive dyes are the most resistant to extraction, because between dye and the cotton fiber, the covalent bond is formed [29,30]. The conventional extraction methods are too weak to cleavage this bond. Reactive dyes can be released from cotton by disrupting the fiber [16]. In order to remove reactive dyes from the fiber, an aqueous solution of a strong base is typically used. The alcohol groups on the glucose unit in the cellulose backbone of cotton act as a weak acid and are ionized under alkaline conditions [29]. During hydrolysis, except for cleavage of the above-mentioned covalent bond, other reactions such as the cleavage of amide bonds and other chemical bonds in the dye molecule are possible. For this reason, the various structural changes can take place, and in many cases, multiple reaction products from a single dye molecule could be produced. The mechanism of the extraction of reactive dyes from cotton, involving dye hydrolysis due to excess NaOH, is shown in Figure 6.

Another method for the separation of a reactive dye from cotton fibers is enzymatic digestion using cellulase solution. However, in this method, a covalent bond between the reactive dye and the cotton fiber is still present, and after enzymatic digestion, a molecule of the dye connected with one or more glucose units is obtained. More details are presented in Section 2.2.2 and Section 2.3.

#### 2.2.2. Extraction of Reactive Dyes from Dyed Cotton Fibers

Reactive dyes are different from other classes of dyes because they form a covalent band with cotton. This makes reactive dyes the most substantive of dyes used on cotton because it provides excellent resistance to washing [31]. However, the extraction of reactive dyes from cotton requires the alkaline hydrolysis of the covalent bond at optimal temperatures or the application of enzymatic digestion.

Table 2 shows the extraction procedure for the isolation of reactive dyes from dyed cotton, which has been described in the literature [3,14,29,32,33,34,35,36,37,38,39].

Method No. 1—As the extraction system, the authors used [32]: sodium–water–poly(vinylpyrrolidone) (PVP), hydrogen bromide, 60% aqueous sulfuric acid, and 1.5% aqueous sodium hydroxide. All solvents were used at 100 °C, with only sulfuric acid used at room temperature. The best results of extraction were reported for sodium hydroxide. Out of 50 reactive dyes, four were successfully extracted using NaOH extraction. However, in the publication, the extraction time was not mentioned [32].

Method No. 2—The material researched was 0.25–5.5 cm^2^ pieces of black cloth (natural fibers such as cotton or wool). Then, 1.5% NaOH was added to the samples, and extraction was carried out for 25 min at 100 °C. Next, 10% (*v*/*v*) methanol was added to the samples [33]. The dry residues (after evaporating the solvent) were dissolved in a 300 µL water/methanol (1:9, *v*/*v*). The samples were diluted with water (1:10). Always, each sample was filtered through 0.45-µm polytetrafluoroethylene (PTFE) filters [33].

Method No. 3—A single fiber 2–15 mm long was extracted in a heat-sealed FEP tube. Then, 5 µL of 0.27 M NaOH and 50% MeOH were added to the research material, and the extraction process was provided at 100 °C (the extraction time was modified depending on the extraction efficiency of the dye). The ca. 20 resin (H^+^ form) particles with 5 µL of methanol/water (1:1) mixture were used to remove NaOH or KOH. After 3–5 min of extraction, the pH-neutralized extract was transferred to a glass microvial and evaporated to dryness [34].

Method No. 4—The authors [29] prepared 10-cm threads of cotton fiber to optimize conditions of macro-scale extraction. The samples were placed into 500-μL glass inserts and put in a 96-well plate system. Solvents were added to the samples using an automated liquid-handling workstation and programmed operations for the specific fiber–dye combinations. The extraction of reactive dyes from cotton was provided using: (1) 29.7% aqueous ammonium hydroxide, (2) 1.5% aqueous solutions of sodium hydroxide, and (3) barium hydroxide. The extraction time and temperature were not presented. Alkaline and alkaline earth cations were removed from the extract using three methods: (1) cation exchange resin (Dowex® HCR-W2, H+ form, spherical beads, 16–40 mesh); (2) solid-phase extraction (SPE) Waters Oasis HLB 6-cm^3^ cartridges were used; (3) the precipitation of barium carbonate by ammonium bicarbonate [29].

Method No. 5—Fibers of 10 mm, 5 mm, and 1 mm lengths, dyed with Reactive Yellow 160, Reactive Blue 220, or Reactive Orange 72 were used as the investigated materials [14]. These fibers were put in 200 μL conical vials, and 50 μL of 0.1875 M sodium hydroxide water solution was added. The vials were sealed to prevent evaporation. The extraction was carried out at 100 °C for 60 min. After that, 25 μL of 0.375 M hydrochloric acid (equimolar to the sodium hydroxide) was added, and subsequently, 25 μL of 10 mM ammonium acetate (adjusted to pH 9.3) was introduced for the dissolution of the reactive dyes [14].

Method No. 6—A 3 mg fabric strip was cut from a 2 × 2 cm textile and immersed in a 5 mL glass vial. Next, 1 mL of 0.15% sodium hydroxide solution was added, and the vial was placed in a heating module, which was subsequently closed. After 1 h at 80 °C, the vial was removed from the module and cooled to room temperature. Next, 30 µL of 1 N hydrochloric acid solution was added for neutralization. The obtained extract was filtered through a 0.2 µm polytetrafluoroethylene filter into an HPLC vial [35].

Method No. 7—A sample 10 mm long fiber was immersed in 10 μL of NaOH solution and cooled at 4 °C for 4 h. After the 4 h cooling time, the NaOH solution was removed. The fiber was washed with acetic acid solution once and with cellulase solution twice. Next, 10 μL of the cellulase solution was added to the rinsed fiber and the sample was mixed in a thermo mixer (Eppendorf Comfort, 50 °C, 550 rpm) for 20 h. Finally, the sample was centrifuged (5000 rpm, 5 min), and 10 μL of methanol was added [36].

Method No. 8—The authors developed three procedures for the isolation of different types of dyes from different types of fibers [37]. One of them was applied for the isolation of reactive dyes from cotton or viscose fibers. In this case, a fiber of 10 mm in length was placed in a vial, and 10 mL of 3 M NaOH was added. After 4 h at 4 °C, the fiber was washed using acetic acid solution (0.5 M) and cellulase solution (1.1 U/mg, 0.01 g in 10 mL of acetic acid solution at pH 5). This procedure was repeated. Next, the rinsed fiber was submerged in 10 mL of cellulase solution and placed in a thermo mixer (Eppendorf Comfort, 50 °C, 550 rpm, 20 h). Finally, after centrifugation (5000 rpm, 5 min), 10 mL of methanol was added to the extract [37].

Method No. 9—The authors based this on the procedure described in Collective Work, European Fibers Group [38]. As the sample, a 2 × 1 cm strip of material was cut from each piece of clothing. From each strip, four thread lengths of 1 cm were prepared. The thread sample was introduced into a 1.5 mL Eppendorf tube and 50 μL of NaOH solution was added. Next, the Eppendorf tube was placed in an ice pocket and put in a refrigerator for 4 h. After 4 h, the NaOH was removed. Firstly, the thread sample was washed using 50 μL of 0.5 M acetic acid solution, and then twice with the application of 150 μL of a 1.6 g/L cellulase solution in acetate buffer (pH 5). After the rinsing procedure, the thread sample was again covered with 150 μL of a cellulose solution and was mixed in a thermomixer for 20 h at 45 °C, 500 rpm. Next, the sample was centrifuged for 5 min at 7000 rpm. Góra and Wąs-Gubała [3] modified the above-described extraction procedure and applied *Aspergillus niger* and *Trichoderma reesei* cellulose. The extraction of reactive dyes using the *Aspergillus niger* cellulase solution was carried out at 50, 55, and 60 °C with the application of a concentration of solutions three and ten times greater than those reported in the literature. A thermomixer was replaced by a water bath and an ultrasound. The same procedure was repeated with the use of *Trichoderma reesei* cellulase. Thus, the extraction conducted was also carried out for eight threads taken from each textile product, and a double volume of each reagent was used. The extract was evaporated to dryness, and the dry residue was dissolved in 150 μL of acetate buffer (pH 5) [3].

Method No. 10—The authors used two methods for the extraction of reactive dyes from fabrics: chemical and enzymatic digestion [39]. Before chemical treatment, the textiles were washed with 2 mL of water, 2 mL of methanol, and 2 mL of acetonitrile (three times with each solvent) in order to remove impurities. The 3 mg fabric samples (three replicates from different sections of the degraded fabrics and one control fabric sample) were placed in 5 mL Fisher glass vials, and 1 mL of 1.5% NaOH solution was added to each vial. Next, the vials were heated at 80 ℃ for 1 h with constant stirring. After cooling down, the extracts were neutralized by the addition of 300 µL of 1 M HCl solution and were filtered through polytetrafluoroethylene (PTFE) syringe filters into HPLC vials. Before enzymatic digestion, 3 mg of the control fabric and degraded samples were prewashed by the application of firstly, 1 mL of water and gently shaken by hand, secondly, 1 mL of methanol, and thirdly, 1 mL of acetonitrile. Finally, the washed fabric samples were dried at room temperature (RT) and were subjected to enzymatic treatment. After prewashing, the 3 mg fabric sample was transferred to a vial. Then, 100 μL of 3 M NaOH solution was added, and the vial was placed in a grip seal bag and in a container with ice and cooled for 4 h. Next, the NaOH solution was removed, and 500 μL of 0.5 M acetic acid was added. After incubation for 1 min, the acetic acid was removed, and 1.5 mL of buffer solution (0.1 M sodium acetate, pH 5 with acetic acid) was added to the extract for a period of 1 min. After this time, the buffer solution was removed, and 1 mL of enzyme solution (90 g of cellulase in 50 mL of buffer) was added. The vial was tightly closed and placed in a shaking bath for 24 h at 50 °C. Finally, the vial was sonicated for 30 s. Next, the extract was filtered and transferred to an HPLC vial [39].

**Figure 7:**
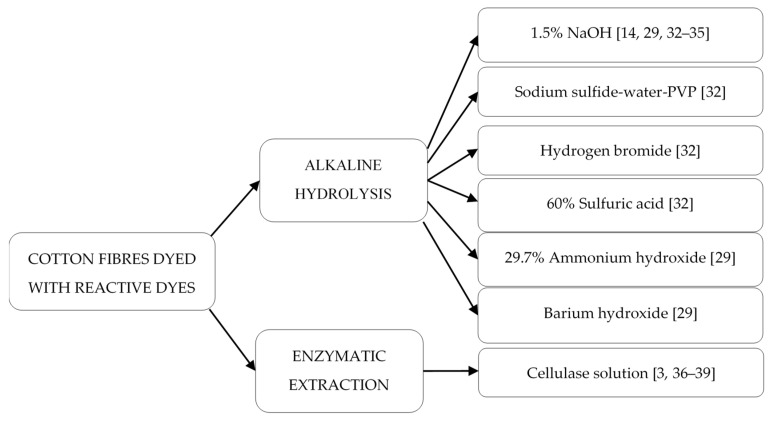
Reagents applied for the isolation of reactive dyes from dyed cotton fibers.

According to the data presented above, the most commonly used method for the isolation of reactive dyes from cotton fibers was chemical treatment [32], especially alkaline hydrolysis [14,29,32,33,34,35], followed by enzymatic extraction (enzymatic digestion) [3,36,37,39] (Figure 7).

The reagent most commonly used for the cleavage of reactive dyes from dyed cotton was 1.5% NaOH [14,29,32,33,34,35], but in a few cases, sodium sulfide-water-poly(vinylpyrrolidone) [32], hydrogen bromide [32], 60% aqueous sulfuric acid [32], 29.7% aqueous ammonium hydroxide [29], and barium hydroxide [29] were also applied. Investigations were performed for such textile materials as a piece of clothing 0.25–5.5 cm^2^ [33]; 3 mg [35,39], 10 cm threads of fiber [29]; and single fibers in a 1–15 mm length range [14]. Alkaline hydrolysis was conducted for 25 min [33] or 60 min [14,35,39]; the applied extraction temperature was 80 °C [35,39] or 100 °C [14,33,34]. Depending on the size of the investigated samples, the volume of NaOH solution was different: 5 µL [34], 50 µL [14], 500 µL [29], and 1 mL [35,39]; the data for other solvents were not presented (Table 2, Figure 7).

The first step of enzymatic extraction was similar to alkaline hydrolysis as presented above [3,36,37,39], with only a different volume of the added NaOH solution: 10 µL [36,37], 50 µL [3], and 100 µL [39]. After this step, the investigated material was usually rinsed in acetic acid and cellulase solution [36] or cellulase solution in acetic acid at pH 5 [37] or cellulase solution in acetate buffer solution at pH 5 [3,39]. Next, cellulase solution was added to the sample and enzymatic extraction was carried out using different procedures: 20 h with stirring (500 rpm) at 50 °C [36,37], 20 h in a water bath or ultrasound at 50, 55, and 60 °C [3], and 24 h in a shaking bath at 50 °C [39]. Investigations were performed for such textile materials as 3 mg of fabric samples [39], threads of a length of 1 cm [3], and 10 mm of fiber [36,37]. Depending on the size of the investigated sample, the required volume of cellulase solution was 10 µL [36,37], 150 µL [3], and 1 mL [39].

To summarize, the extraction of reactive dyes from cotton was mainly carried out by alkaline hydrolysis and enzymatic extraction. The best results were obtained for 1.5% NaOH (alkaline hydrolysis) and cellulase solution (enzymatic extraction). However, alkaline hydrolysis was shorter than the enzymatic extraction.

#### 2.2.3. Extraction of Unknown Reactive Dyes from Dyed Cotton and Viscose

The extraction procedures presented above are dedicated for cotton fibers dyed with reactive dyes. However, most textiles can be and are dyed with multiple dyes. This fact may be useful to forensic scientists, but it also may lead to complications in the analysis of dyes in unknown samples. Different extraction systems and schemes for the classification of different dyes have been proposed in the literature. All of the systems and schemes have the same goal; they are intended to lead to the identification and classification of dyes. Among them, Laing and co-workers [40] for the first time proposed a scheme for the identification of reactive dyes on cotton. It was an exclusionary procedure. In this procedure, reactive dyes were not extracted by organic solvents, but when the fibers were treated with a reducing agent (sodium dithionite in sodium hydroxide), the original dyes, if azo in nature, were irreversibly decolorized. Thus, this procedure allows azo reactive dyes to be distinguished from other dye classes, but the identification of reactive dyes by TLC was impossible [40].

**Figure 8:**
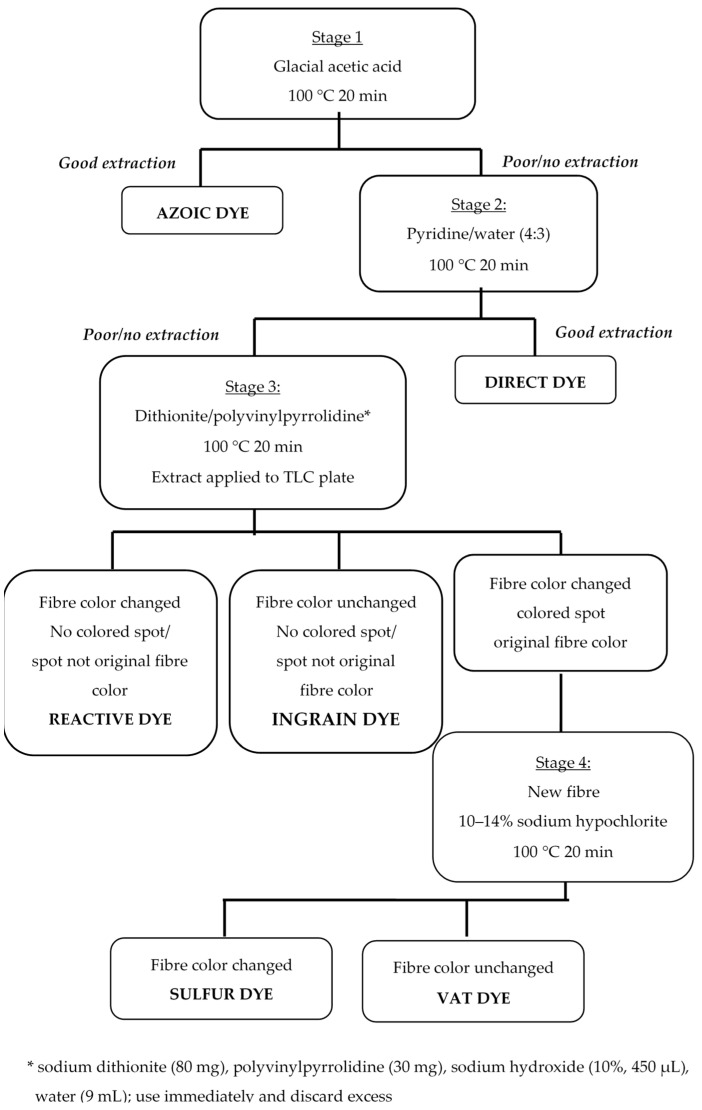
Classification scheme for the extraction of dyes from cotton and viscose fibers. Reprinted from Lewis S.W., Identification of Textile Fibers/Chapter 11: Analysis of dyes using chromatography, Pages 203–223., Copyright (2009), with permission from Elsevier [41].

Experts from the former Forensic Science Service of England proposed a general, comprehensive, and widely used extraction scheme for the isolation of different types of dyes from dyed fibers [16]. It was also presented by Lewis [41], and this scheme is shown in Figure 8.

The extraction of dyes from fibers is a very simple process. A single fiber should be put in a glass tube, which should be sealed at one end. Around 10 μL of solvent should be added to the tube. The tube should be sealed and heated in the oven according to the time and temperature described in the extraction procedure. Details of the extraction scheme for dyes isolated from cotton and viscose fibers are shown in Figure 8 [41].

### 2.3. Identification of Dyed Cotton Fibers for Forensic Purposes Based on Chromatographic Techniques

**Figure 9:**
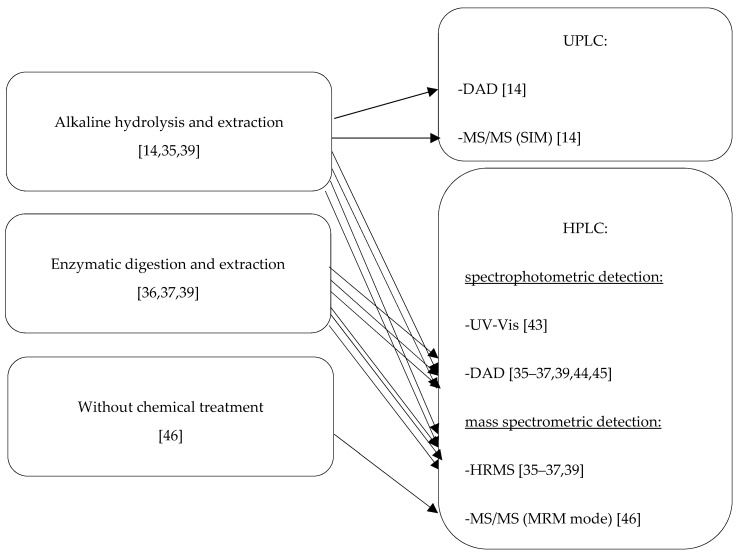
Chromatographic methods applied for the identification of extracted reactive dyes for forensic purposes.

Fiber traces, being the subject of forensic analyses, are usually no more than a few millimeters long and they usually contain 2 to 200 ng of dye [42]. For this reason, sensitive analytical methods must be applied for the identification of reactive dyes isolated from dyed cotton fibers. Table 3 and Table 4 show the methods developed for the identification of reactive dyes based on chromatographic techniques [14,35,36,37,39,43,44,45,46]. A description of the chromatographic conditions is included in Table 3; the selected qualification and quantification parameters are in Table 4. A general scheme of the identification of extracted reactive dyes for forensic purposes based on chromatographic techniques is presented in Figure 9.

#### 2.3.1. Chromatographic Conditions

High-performance liquid chromatography (HPLC) coupled with spectrophotometric detectors such as UV-Vis [43] and diode array (DAD) [35,36,37,39,44,45], as well as coupled with high-resolution mass spectrometric (HRMS) [35,36,37,39] and tandem mass spectrometric (MS/MS) detectors [46] was the most often used technique for the identification of reactive dyes for forensic purposes (Figure 9, Table 3 and Table 4). Usually, the HPLC-MS apparatus was equipped with both detection systems [35,36,37,39] (Figure 9; Table 3 and Table 4). Chromatographic analyses were usually performed using reverse-phase C18 columns [35,39,43,44,45,46] of a length from 50 to 150 mm. On the other hand, Carey et al. and Schotman and co-workers applied a Grom-sil 120 ODS-5 ST chromatographic column with a length of 150 mm [36,37]. In most cases, the chromatographic separation of reactive dyes was carried out under gradient programs using such mobile phases as an ammonium formate aqueous phase and formic acid in water (pH 4): a ratio of 70/30 methanol/acetonitrile organic phase [35]; ammonium acetate in water/methanol (95/5): ammonium acetate in acetonitrile/methanol (50/50) [36,37]; ammonium formate and formic acid in water (pH 4): methanol/acetonitrile (70/30) [39]; ammonium acetate in water/acetonitrile (90/10) (pH 6): ammonium acetate in water/acetonitrile (10/90) [44,45]; acetic acid with water/acidified acetonitrile [46]. In one case, the isocratic conditions using a 47:53 *v/v* mixture of acetonitrile and ammonium acetate buffer containing trimethylammonium bromide (CTAB) as an ion-pairing agent was applied [43]. The mobile phase flow rate was in the range of 0.3 mL/min to 0.8 mL/min; the time of analysis was from 9.5 [39] to 78 min [37]; the injection volume was 10 or 20 μL.

The next chromatographic technique applied for the identification of reactive dyes for forensic purposes was ultra-performance liquid chromatography (UPLC) coupled with DAD and MS/MS detectors [14] (Figure 9; Table 3 and Table 4, Method No. 1). The separation of analytes was done using a C18 column with a length of 50 mm, and 10 mM of ammonium acetate (pH 9.3) and acetonitrile as mobile phases in gradient conditions. The mobile phase flow rate was 0.4 mL/min, the time of analysis was 5 min, and the injection volume was 10 μL [14].

#### 2.3.2. Qualification and Quantification Analysis of Extracted Reactive Dyes for Forensic Purposes

As mentioned in Section 2.2, the necessity to cleavage the covalent bond formed between the dye and cotton fibers using alkaline hydrolysis or by enzymatic digestion results in various structural changes of reactive dyes and in the formation of multiple reaction products from a single dye molecule. For this reason, prior to chromatographic analyses, the synthesis and the study of partially and completely hydrolyzed forms of selected reactive dyes is very useful. The knowledge of the structures of their conjugates with glucose units formed during enzymatic digestion is also very important because they could be used as standards during the qualification and quantification analysis of reactive dyes extracted from dyed cotton fibers.

##### Synthesis of Hydrolyzed and/or Enzymatic Digestion Dye Standards

Nayar and Freeman (2008) analyzed the commercial and hydrolyzed forms of six reactive dyes (C.I. Reactive Red 2, Reactive Red 24:1, Reactive Orange 72, Reactive Blue 19, C.I. Reactive Blue 4, C.I. Reactive Red 120) using negative ion fast atom bombardment (FAB) and negative ion electrospray (ES) mass spectrometry [47]. These dyes were selected as representatives of the structural types of currently used reactive dyes. Negative ion FAB and ES mass spectrometric analyses have been used to determine the level of hydrolysis following the dyeing of cotton with reactive dyes [47].

The synthesis of hydrolyzed dye standards of C.I. Reactive Blue 19 was also performed by Sultana et al. [35]. The obtained hydrolyzed dye standard (RB19-OH) was characterized via DAD and high-resolution MS, and it was used for the quantification and qualification of dyes on biodegraded samples.

Feng et al. [39] also decided to synthesize hydrolyzed dye standards and enzymatic digestion dye standards having cellobiose units of four commonly used reactive dyes, C.I. Reactive Black 5, C.I. Reactive Red 198, C.I. Reactive Blue 49, and C.I. Reactive Orange 35, in order to use them as standards for comparison in the study of the biodegradation of reactive dyes on cotton jersey fabrics buried in soil. The structures of the degradation products were determined using the HPLC-HRMS technique [39].

##### Identification and Quantification of Extracted Reactive Dyes for Forensic Purposes

Hoy demonstrated successful alkaline hydrolysis and extraction of reactive dyes from single 10 mm, 5 mm, and 1 mm cotton fibers dyed with Reactive Yellow 160, Reactive Blue 220, or Reactive Orange 72, and they analyzed the obtained extracts using the UPLC-DAD-MS/MS technique (Table 3 and Table 4, Method No. 1) [14] (Figure 9). UPLC-DAD and UPLC-(ESI)MS/MS analyses of standard solutions of reactive dyes and extracted (treated with NaOH) reactive dyes confirmed that additional reactions occur during the extraction process. For example, the UPLC-DAD chromatogram for standard Reactive Orange 72 showed a single peak corresponding to the dye with an absorbance maximum of 478 nm, whereas the UPLC-DAD chromatogram for extracted dye showed two peaks, both of which increased in retention, and the main peak had an absorbance maximum of 473 nm. Hoy suggested that both peaks corresponded to the dye and one of them corresponded to the incomplete base hydrolysis of Reactive Orange 72. An analysis of the Reactive Orange 72 dye standard using UPLC-MS/MS confirmed that Reactive Orange 72 is actually a mixture of derivatives of the dye, which are characterized by *m/z* values at 572 (retention time (RT) 13.92 min), *m/z* 492 (RT 14.14 min), *m/z* 474 (RT 15.87 min) and *m/z* 417 (RT 14.47 min). On the other hand, the UPLC-MS/MS analysis of Reactive Orange 72 treated with NaOH confirmed the presence of only one product at *m/z* 450 (RT 13.24 min); the second peak observed during the UPLC-DAD measurement did not appear. The Reactive Yellow 160 standard was also a mixture of derivatives at *m/z* 652 (RT 16.61 min), *m/z* 572 (RT 17.50 min), *m/z* 554 (20.77 min), and *m/z* 614 (RT 19.92 min); whereas Reactive Blue 220 was not observed in UPLC-MS/MS. The amount of dye on each fiber was determined based on the peak area on the chromatogram acquired for each dye at the maximum wavelength (405, 610, and 478 nm, respectively for Reactive Yellow 160, Reactive Blue 220, and Reactive Orange 72) and comparison with standard dye mixtures. The limits of detection (LOD) of the investigated reactive dyes using UPLC-MS/MS were in the range from 3 to 83 pg (based on 10 µL injections), confirming the possibility of performing quantitative analysis of 1 mm fiber extracts. The proposed UPLC-DAD method gave the detection limits of 0.33–1.42 ppb and the quantitation limits of 1.00–4.30 ppb. Trace fiber extractions confirmed the possibility of the detection and quantification of 1–10 mm extracts of Reactive Yellow 160. The obtained UPLC-MS/MS results for Reactive Orange 72 and Reactive Yellow 160 suggested that many reactive dyes could be mixtures of multiple compounds [14].

Sultana and co-workers investigated C.I. Reactive Blue 19 (RB19) extracted from cotton fabrics biodegraded in soil in laboratory conditions over intervals of 45 and 90 days using the HPLC-DAD-HRMS method [35]. High-resolution mass spectrometric detection (ESI(-)-(Q-TOF) was applied to characterize the isolated dye degradation products; DAD detection was used to quantify the intact dye and the degradation product isolated from the fabric samples. As mentioned in this section, prior to chromatographic analysis, the hydrolyzed dye standard (RB19-OH) was synthesized and characterized via DAD (λ_max_ at 620 nm, RT 4.6 min) and high-resolution MS (theoretical *m/z* value 501.0432; value monitored in the mass chromatogram *m/z* 501.0432) [35]. The DAD analysis of the obtained extracts at 620 nm confirmed the presence of two compounds on biodegraded cellulosic fabrics: RB19-OH at the RT of 4.6 min, and the desulfonated degradation product formed after losing an –SO_3_ group from RB19-OH at the RT of 5.2 min (the last signal was observed also during HPLC-HRMS as the deprotonated singly charged molecule at *m/z* 421.0858). Thus, the isolated dye was successfully quantitatively analyzed via the HPLC-DAD method by synthesizing a hydrolyzed form of the dye and creating calibration curves (limit of quantification 0.4 ± 0.2 μg/mL). Moreover, the HPLC-HRMS analysis showed that the degradation product was formed by losing an −SO_3_ group from the intact hydrolyzed form of the dye [35].

The identification of Reactive Orange 16, Reactive Yellow, and Reactive Red 120 extracted from cotton fibers (Table 3 and Table 4, Method No. 3) was based on retention times, and the mass accuracy was recorded using a high-resolution MS detector (deviation generally < 2 ppm) [36]. The HPLC analyses with the application of both detector systems (DAD, MS) were performed for each standard dye solution and for each dye extracted from a fiber, with a continuous record of acquired spectra. In this way, ample data were recorded. The absorption spectra recorded for a standard dye solution in the range of 200–800 nm proved that λ_max_ for Reactive Orange 16 is 494 nm, for Reactive Yellow 145 λ_max_ is 417 nm, and for Reactive Red 120 λ_max_ is 540 nm. In each case, the chromatograms obtained for pure standard dyes and those acquired after the hydrolysis of dyed fibers using the cellulase method were compared. The RTs observed for the powder dye references and extracted reactive dyes were different. For example, the RTs of pure Reactive Orange 16 were 19.4 min (*m/z* 572.009800) and 24.8 min (*m/z* 474.04242); whereas for Reactive Orange 16 fiber, the RTs were 17.6 min (*m/z* 774.14806) and 19.7 min (*m/z* 816.15863), respectively. This means that the recovered dye was still linked to one or more cellulose units and thus differed from the chemical structure of the unreacted dye. This altered the RT and the observed molecular mass. For example, the signal at *m/z* 816.15863 observed for Reactive Orange 16 fiber was attributed to the structure containing the dye molecule connected to two cellulose units. The LODs were established as the minimum concentration of dissolved dye powder (μg/L) and the minimum fiber length required to identify the dye. For example, the LOD values determined using the DAD detector established for Reactive Orange 16 were 37.3 μg/L and 0.06 mm, while for the MS detector, they were 4.2 μg/L and 0.011 mm, respectively [36].

Schotman and co-workers identified dyes present on fibers or textiles submitted to forensic examination using the HPLC–DAD–MS method (Table 3 and Table 4, Method No. 4) [37]. Among seven investigated samples (case samples), four were made from cotton. Reactive Orange 122 was detected in the sample of case 2, while Reactive Orange 195 was detected in the sample of case 3. Usually, one or more of the dyes were identified in the obtained extracts, and the number of identified dyes was from a few to around 50 in case 8. Such data as the retention time, DAD spectrum, and mass spectrum allowed a useful “fingerprint” of the dye to be provided, which could at least be very useful to compare fibers from a known source with recovered traces [37].

Feng at al. applied the HPLC-DAD-HRMS method for the determination and characterization of products obtained from four reactive dyes (C.I. Reactive Black 5 (RBlk5), C.I. Reactive Red 198 (RR198), C.I. Reactive Blue 49 (RB49), and C.I. Reactive Orange 35 (RO35)) present on cotton jersey fabrics subjected to biodegradation in soil (Table 3 and Table 4, Method 5) [39]. The optimized chemical and enzymatic procedures allowed the cotton fabric to be digested, and they removed RBlk5, RR198, and RB49 from the dyed material. As mentioned in this section, hydrolyzed reactive dyes and reactive dyes having cellobiose units were synthesized and used as standards for comparison in this study. The determination of the structures of the biodegraded products, based on exact mass measurements supported by the DAD measurements (retention time and absorbance spectra), allowed the possible degradation pathways of the reactive dyes in degraded cotton fabrics to be proposed [39].

Zotou and co-workers investigated the hydrolysis kinetics of two reactive fluorotriazinic dyes (Cibacron Yellow F-4G and Cibacron Blue F-R) separately and in a 1:1 mixture, in the presence as well as in the absence of cotton, on a laboratory scale using reversed-phase ion-pair HPLC (Table 3 and Table 4, Method No. 6) [43]. The two forms of the dyes, hydrolyzed and non-hydrolyzed, were separated satisfactorily (different RTs). However, when these two dyes were studied as a mixture, the hydrolyzed Blue co-eluted with the non-hydrolyzed Yellow. For this reason, in order to obtain complete separation, the flow rate of the mobile phase during the HPLC analysis has to be decreased from 0.8 to 0.6 mL/min [43].

Chemchame and co-workers also studied the hydrolysis of reactive dyes (Table 3 and Table 4, Methods No 7 and No. 8) [44,45]. In these studies, four standard dye solutions of heterobi-functional reactive dyes containing monochlorotriazine/β-sulfatoethylsulfone (C.I. Reactive Red 195, C.I. Reactive Yellow 145, and C.I. Reactive Blue 221) were prepared and subjected to hydrolysis using NaOH concentrations fixed at pH 11 at 98 °C for 90 min [44] or using NaOH concentrations fixed at pH 10 at 60 °C for 30 min [45]. Among the three methods tested for quantifying unfixed forms of bi-functional reactive dyes used in dyeing cotton fibers (spectrophotometric approach, colorimetric approach, and HPLC technique), the last one was the most useful to identify and quantify the hydrolyzed and inactive forms of dye in a residual soaping bath.

Hu and co-workers developed a sensitive HPLC-MS/MS method using Multi Reaction Monitor (MRM) mode for the analysis of different fiber dyes, among them for the analysis of cotton fibers dyed with reactive dyes (Table 3 and Table 4, Method No. 9) [46]. Unfortunately, in this study, the extraction of reactive dyes from cotton fibers was not presented (such information was presented only for disperse dyes extracted from polyester fibers). The HPLC-MS/MS analyses were performed both in positive ion mode (the molecular ions [M + H]^+^ and [M − Cl]^+^ were observed) as well as in negative ion mode ([M − H]^−^, [M − Na]^−^, and [M − 2Na]^2−^ were observed). Reactive Orange 16 (RO16) (calculated molecular mass at *m/z* 617.54) produced a molecular ion in the form of [M−2Na]^2−^ at *m/z* 285.4, which was subjected to fragmentation to an ion at *m/z* 236.85 and an ion at *m/z* 264, as observed in MRM mode. The RT of reactive dye RO16 was 5.09 min; the limit of detection during MS/MS measurements was 2.10 ng/mL [46].

### 2.4. Examination of Dyed Textile Fibers for Forensic Purposes Based on Routinely Used Spectroscopic Techniques

Routine methods of identifying and comparing textile fibers in forensic science are based on the principles of microscopy and spectroscopy. Information derived from microspectrophotometry and vibrational spectroscopy investigations has contributed to the benefit of forensic examinations of dyed textile fibers. This is emphasized by examples taken from real case studies and targeted scientific research in this field [16,48].

UV–visible microspectrophotometry (UV-Vis MSP) is used for objective observations of colored fibers because it is nondestructive, repeatable, and unlike other methods that require the extraction of the dye, it involves little sample preparation. Despite this, it is rather used for identifying and comparing the spectral characteristics of a sample, not identifying particular dyes or mixtures of dyes [49].

The discrimination of single cotton fibers dyed with reactive dyes of the same manufacturer, as well as the possibility of assessing the concentration of a dye in examined fibers were verified with the use of UV-Vis MSP [50]. Woven cotton fabrics dyed with different concentrations of one-compound reactive dyes (the commercial name Cibacron^®^) were examined, and the results obtained indicated that all of the analyzed samples were distinguishable from each other with the use of this technique. The detection limit was 0.18% of the concentration of a dye in the textile sample. However, the authors observed intra-sample and inter-sample variation, as well as the dichroism effect.

Subsequently, the same authors presented an assessment of the applicability of UV-Vis MSP together with Raman spectroscopy in the examination of textile fibers dyed with mixtures of synthetic, also reactive dyes [51]. The MSP study conducted in the 200 to 800 nm range and three types of excitation sources, 514, 633, and 785 nm, used during Raman examinations, were applied for the examination of single cotton fibers, which were dyed with binary or ternary mixtures of reactive dyes. UV–Vis MSP showed limited possibilities for the discriminatory analysis of cotton fibers dyed with a mixture of reactive dyes, where the ratio of the concentration of the main dye used in the dyeing process to the minor one was higher than four. The results show that the capability of distinguishing dye mixtures was similar for both spectroscopic methods used.

Buzzini and Massonnet evaluated the potential and limitations of Raman spectroscopy on a broad range of fiber types and colors, including those containing reactive dyes [52]. For fiber samples collected from 180 textiles, the results obtained using Raman spectroscopy were compared to those collected with the use of traditional methods of textile fibers examination, i.e., bright field, double polarization, fluorescence and comparison microscopy, UV-Vis MSP, and TLC. This study presented that Raman spectroscopy can play a complementary role in the routine analytical sequence of forensic fiber analysis to detect and identify the dye composition. Combining data obtained with several laser wavelengths allowed for the further discrimination of pairs previously indistinguishable with the use of light microscopy and UV-vis MSP. Despite the lower discriminating power calculated for Raman data when single laser wavelengths were considered, additional discriminations were observed. It was concluded that an instrument equipped with several laser lines is necessary for efficient use [53].

Was-Gubala and Machnowski [54] confirmed the difference between cotton and regenerated cellulose fibers resulting from variations in the degree of polymerization and supramolecular structure during Raman research [54]. The examined fibers were dyed with eight reactive dyes, among others, and their spectra were obtained with the use of three excitation sources: 514, 633, and 785 nm. For more than 80% of the obtained spectra, the presence of dye bands was confirmed. Bands originating from cotton and viscose were usually predominant in the spectra of fibers with a lower dye concentration, while in the other case, the majority of the bands originated from the dye. However, the dye concentration in cotton and viscose fibers did not directly influence the interrelationships between the intensity of characteristic dye bands and their concentration in the fiber samples in every case.

Techniques that have increased Raman sensitivity in the last decade are surface-enhanced Raman spectroscopy (SERS) and surface-enhanced resonance Raman scattering (SERRS). However, they are more complex and require more experience in sample preparation than Raman spectroscopy itself; therefore, there are not many reports of their use in forensic practice, for example for the examination of reactive dyes in cotton fibers yet. Nevertheless, in other scientific fields, they are used for dyes and textiles research [55,56,57,58,59,60].

While the use of infrared microscopy is almost natural to determine the composition of fibers, its use to identify fiber dyes may be appropriate in highly colored fibers and is likely to be below the pale fiber detection limits [61]. This stems from the lack of sensitivity of infrared absorbance to components that represent less than approximately 5% of a sample and the characteristically low level of dye concentration in most textile fibers, which is also those dyed by reactive dyes. Grieve reported more specific contributions of known dyes to the region of the infrared spectra of acrylic fibers [62]. Diffuse reflectance infrared Fourier transform spectroscopy (DRIFTS) has been more successful in characterizing fiber dyes, and the reactive dyes were discriminated on cotton by use of this technique [63].

## 3. Comparison of Chromatographic Methods for the Identification of Dyed Textile Fibers for Forensic Purposes with a Spectroscopic Technique

**Figure 10:**
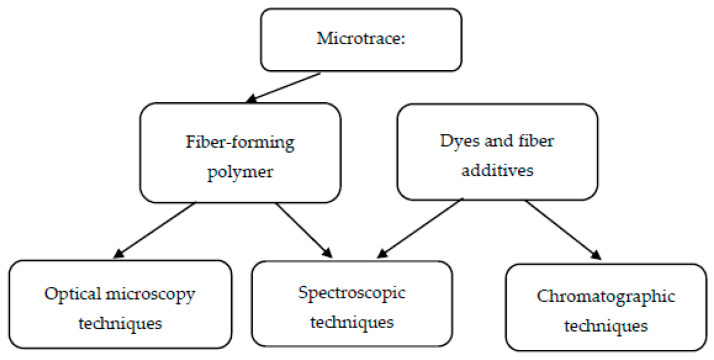
Scheme of forensic examination of textile fibers.

Forensic examinations have to give as much evidential information as possible. The identification and comparison studies of microtraces as single dyed fibers for forensic purposes are based on microscopic, spectroscopic, and chromatographic techniques [6] (Figure 10).

The first step in the forensic laboratory is the investigation of dyed textile fibers using optical microscopy. Optical studies are based on different techniques. A microscopic image of a fiber gives information about its physicochemical structure. Fibers can be investigated mainly by stereoscopic microscopy with reflected light, biological microscopy with transmitted light, polarized light microscopy, and fluorescence microscopy with UV light. For example, scanning electron microscopy can be used to study damage to textile fibers, but as the obtained image is black and white, this technique is not suitable for the study of fiber dyes.

Following the selection of samples and after testing them by microscopy, other methods have to be chosen for further investigations of the fibers, and the choice of such methods usually depends on the laboratory equipment. The most often applied methods are spectroscopic techniques, and the least ones used are chromatographic ones.

All spectroscopic techniques (FTIR, Raman Spectroscopy, and UV-Vis MSP) are nondestructive, which is very important in forensic science. Spectroscopic techniques often give spectra with information about dyes, fiber-forming polymers, or fiber–dye mixtures. The results depend on the applied spectroscopic techniques (Raman Spectroscopy, UV-Vis MSP, or FTIR) and on the analysis conditions.

Chromatographic techniques have a destructive character, but they can give a lot of information about the dyed fibers as evidence. Identification is based on several parameters: retention times, mass spectra, or the selected, characterized *m/z* values of dyes. These parameters allow for the identification and comparative research between the dyes from evidential and comparative materials.

The next parameters that are different for spectroscopic and chromatographic techniques are the time of sample preparation and the time of analysis. Both times are longer for chromatographic than spectroscopic techniques.

In chromatographic techniques, the investigation could be divided into three main steps: (1) extraction of the dye/s from the fiber sample; (2) analysis of the obtained extract by the chromatographic method; (3) analysis of the obtained data. The sample preparation step requires the use of additional reagents and some special equipment, but the chromatographic conditions applied for the identification of individual types of dyes are constant. The identification of dyes is based on chromatographic (retention times) and mass spectrometric (*m/z* values) data.

In spectroscopic techniques, a fiber/a thread is the sample, but the conditions for the analysis must be determined individually.

Summary: the first step-microscopic investigation of the target dyed textile fibers is always the same. After this step, other methods, e.g., both spectroscopic and chromatographic, have to be chosen for further examination of the textile material. This choice of methodology usually depends on the laboratory equipment and such factors as the form and volume of the evidence, the type of textile, the type of dyes, etc. Both spectroscopic and chromatographic methods have advantages and disadvantages regarding the identification of dyed cotton fibers. The results and how they can be interpreted should be discussed within the perspective of previous studies and of working hypotheses. The findings and their implications should be discussed in the broadest context possible. Future investigations may also be proposed.

## 4. New Challenges in Identifying Cotton Fibers Dyed with Reactive Dyes for Forensic Purposes

The examination of dyed cotton fibers in forensic laboratories (e.g., in the form of microtraces found at a crime scene) usually consists of comparing them with fibers originating from a known source (e.g., the suspect’s clothing) or determining their potential source (e.g., knitted or woven fabric) or comparing them with other evidential fibers (e.g., revealed under the victim’s fingernails). The developed chromatographic methods, especially with mass spectrometric detection, are very sensitive, and they are very suitable for the comparison of evidential fibers to known fibers, i.e., whether the evidential fiber has the same dye as the known fiber [46]. In order to compare evidential fibers to known ones, it is necessary to establish an HPLC-MS/MS dye analysis database. When presenting the sequence for examining dyed fibers for forensic purposes, it is first necessary to identify the type of fiber, and then, knowing the classes of commonly used dyes for a given type and color of fibers, narrow down the range of possible dyes. Combined with the peak time and molecular and product ions in the database, a comparison between unknown fibers can be achieved.

Many scientists and practitioners are working on the construction of an analysis database of Raman spectroscopy and HPLC-MS/MS for common dyes. However, the development of the textile and dyes industry is so great that new information has to be continuously entered into such a database.

The chromatographic methods described above were shown to be suitable for the analysis of reactive dyes extracted from dyed cotton fibers. The high sensitivity and wide applicability make it possible to implement this methodology in routine case work. However, challenges associated with the compatibility of these chromatographic methods with fiber dye extraction solvent systems remain to be overcome. In addition, it is important to build a database of reactive dye mass spectra for the better detection of unknown fibers.

## 5. Conclusions

In forensic laboratories, the first step of the identification and comparison of fibers as microtraces is the use of optical microscopy techniques. An important feature in such research is the fiber color. Subsequently, if the colors of the evidential and comparative materials are similar, studies are carried out using UV/VIS MSP and Raman spectrometry, and occasionally by infrared spectrometry, mass spectrometry (MS), high-performance liquid chromatography (HPLC) or by using a combination of the last two techniques. Cotton fibers are natural and they are rather difficult to distinguish on the basis of morphological characteristics. For this reason, their color and consequently dye composition are often their only characteristic features. To our knowledge, this is the first review paper in which (1) the possibility of the application of chromatographic methods for the analysis of cotton fibers dyed with reactive dyes for forensic purposes was presented; (2) a chromatographic approach was compared with a spectroscopic one; and (3) the advantages and limitations of both methodologies were shown. Among chromatographic methods for the identification of reactive dyes for forensic purposes, the most often used is the HPLC-HRMS system, while among spectroscopic ones, UV-Vis MSP dominates. Chromatographic techniques can give much more information about the dyed fibers as evidence (identification is based on retention times, mass spectra or the selected, characterized *m/z* values of dyes) in comparison to spectroscopic ones. On the other hand, spectroscopic techniques are nondestructive, which is very important in forensic science. They often give spectra with information about dyes, fiber-forming polymers, or fiber–dye mixtures. The presentation of the above-mentioned procedures may help to choose the best analytical method for the effective characterization of cotton fibers dyed with reactive dyes. This knowledge could be useful for forensic experts and could help them to avoid mistakes. Moreover, this information/these data could be very interesting also for scientists working in food, cosmetics, or pharmaceutical/medical sciences.

## Figures and Tables

**Table 1:** Examples of reactive dyes with different reactive groups.

Reactive Group	Chemical Structure
Monochlorotriazine	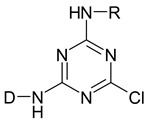
Dichlorotriazine	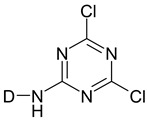
Dichloropyrimidine	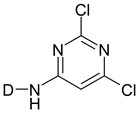
Dichloroquinoxaline	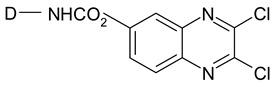
Aminochlorotriazine	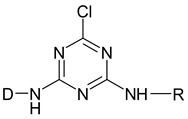
Monofluorotriazine	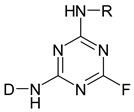
Fluorochloropirymidine	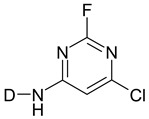
Aminofluorotriazine	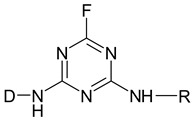
Vinyl Sulphone	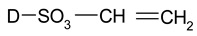
Sulphatoethylsulphone	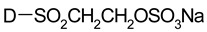

**Table 2:** Overview of extraction procedure for isolation of reactive dyes from dyed cotton fibers described in the literature.

			Extraction Procedure					
No. Method	Fiber/Dyes	Cleavage of Covalent Bonds of Reactive Dyes With Functional Groups on the Cotton Fibers	Solvents	Fiber Length	Volume	Temperature	Time/Additional Information	Lit.
**1**	Dyes extracted from manufacturers’ pattern cards and casework materials (50 different reactive dyes)	Hydrolysis	Sodium sulfide–water–poly(vinylpyrrolidone) (PVP) Hydrogen bromide 60% aqueous sulfuric acid 1.5% aqueous sodium hydroxide	n.d.	n.d.	Sulfuric acid at room temp.; other solvents at 100 °C	n.d.	[32]
**2**	Black cotton/Names of reactive dyes not presented	Alkaline hydrolysis	1.5% NaOH; 10% MeOH (*v*/*v*)	0.25–5.5 cm ^2^ of black cloth (cotton)	n.d.	100 °C	25 min Most of the solvent was evaporated and the precipitate was dissolved into 300 µL of water–methanol (1:1, *v*/*v*). Next, the samples were diluted with water (1:10) and filtered through 0.45-/zm PTFE filters and analyzed using capillary zone electrophoresis with UV detection	[33]
**3**	Cotton 35% and Polyester 65%/black/Cibacron (reactive dyes) Dianix (disperse dyes) Cotton/brown/ Brown A-96138, reactive dyes: 1.66% Procion Yellow H-EXL 0.62% Procion Crimson H-EXL 1.72% Procion Blue H-ERD Cotton/marine/ Marine A-9514, reactive dyes: 0.96% Procion Yellow H-E4R 1.6% Procion Red H-EXL 5.16% Procion Navy H-ER	Alkaline hydrolysis	0.27 M (1.5%) NaOH; 50% MeOH	A single fiber of 2–15 mm	5 µL	100 °C	The extraction time varies depending on the extraction efficiency of the dye(s). Resin H(+) form was used to remove NaOH. Next, the samples were analyzed using micellar electrokinetic capillary chromatography	[34]
**4**	Cotton dyed fabrics and standard dyes: Reactive Blue 21, Reactive Yellow 160, Reactive Orange 72, Reactive Blue 19, Reactive Yellow 176, Reactive Violet 5, Reactive Black 5, Reactive Blue 250, Reactive Red 198, Reactive Blue 220, Reactive Red 180, Reactive Red 239/241	Alkaline hydrolysis in automated extraction system	Solvent systems: 29.7% aqueous ammonium hydroxide, 1.5% aqueous solutions of sodium hydroxide, barium hydroxide	10-cm threads of fiber (yarns consisting of a bundle of twisted fibers)	500 µL	n.p.	n.d. Three methods to remove alkaline and alkaline earth cations from the extract: (1) Cation exchange resin using Dowex^®^ HCR-W2, H+ form, spherical beads, 16–40 mesh; (2) Solid-phase extraction (SPE) using Waters Oasis HLB 6-cm^3^ cartridges. (3) Precipitation reactions using ammonium bicarbonate to precipitate barium carbonate. Next, the samples were analyzed using capillary electrophoresis	[29]
**5**	Reactive Yellow 160, Reactive Blue 220, Reactive Orange 72	Alkaline hydrolysis	1.5% NaOH	Fibers of lengths 10 mm, 5 mm, and 1 mm	50 µL	100 °C	1 h The resulting solution was then treated with 25 µL of 0.375 M hydrochloric acid (equimolar with the sodium hydroxide), 25 μL of 10 mM ammonium acetate adjusted to pH 9.3 to neutralize any excess sodium hydroxide remaining prior to UPLC-DAD-MS/MS	[14]
**6**	Cotton/Reactive Blue 19	Alkaline hydrolysis	0.15% sodium hydroxide solution	3 mg fabric strip was cut from 2 × 2 cm fabric samples	1 mL	80 °C	1 h After 1 h, the vail was cooled to room temperature, neutralized with 30 μL of 1 N hydrochloric acid solution, filtered with a 0.2 μm polytetrafluoroethylene filter and analyzed using HPLC-DAD-HRMS	[35]
**7**	Cotton/Reactive Yellow 145 Cotton/Reactive Red 120 Cotton/Reactive Orange 16	Enzymatic extraction	10 μL of NaOH solution; 4 °C, 4 h. Next, the NaOH solution was removed and the fiber was rinsed in acetic acid solution and twice in cellulase solution. Then, the fiber was submerged in 10 μL of cellulase solution and mixed in a thermo mixer (Eppendorf Comfort, 50 °C, 550 rpm).	10 mm piece of fiber	10 μL	50 °C	20 h Afterward, the samples were centrifuged (5000 rpm, 5 min), and 10 μL of methanol was added. Next, the samples were analyzed using HPLC-DAD-MS	[36]
**8**	Cotton/Reactive Orange 122 Cotton/Reactive Red 195	Enzymatic extraction	10 μL of NaOH solution (3 M, 4 °C, 4 h. Next, the NaOH solution was removed and the fiber was rinsed twice in acetic acid solution (0.5 M) and in cellulase solution 0.01 g in 10 mL acetic acid solution at pH 5). Then, the fiber was submerged in 10 μL of cellulase solution and mixed in a thermo mixer (Eppendorf Comfort, 50 °C, 550 rpm)	10 mm of fiber	10 μL	50 °C	20 h Afterward, the samples were centrifuged (5000 rpm, 5 min), and 10 μL of methanol was added. Next, the samples were analyzed using HPLC-DAD-MS	[37]
**9**	21 pieces of red clothing, of a similar shade of color, made solely of cotton or with addition of other type of fibers polyesters, modal, elastane/Names of reactive dyes not presented	Enzymatic extraction Cellulase from Aspergillus niger Trichoderma reesei ATCC 26921	Basic procedure: 50 μL NaOH solution for 4 h at 4 °C. Next, the fibers were washed first in 50 μL of 0.5 M acetic acid solution and then twice in 150 μL of a 1.6 g/dm3 cellulase solution in acetate buffer (pH = 5). Next, the fibers were again covered with 150 μL of a cellulose solution Concentration of cellulase solutions were three and ten times greater than in basic procedure Concentration of cellulase solutions were three and ten times greater than in basic procedure and double volume of reagents. The extraction was carried out in bath water and the ultrasounds	4 (1) and 8 (2) threads of a length of 1 cm	150 μL	50 °C; 55 °C, 60 °C, respectively	20 h After that centrifugation 5 min, 7000 rpm. Next, the samples were analyzed using TLC coupled with VSC	[3,38]
**10**	Degraded Cotton/Reactive Red 198 Degraded Cotton/Reactive Black 5 Degraded Cotton/Reactive Blue 49 Degraded Cotton/Reactive Orange 35	Two methods: Chemical Treatment Method Before treatment, the degraded fabrics were washed three times with different solvents (2-mL water, 2-mL methanol, and 2-mL acetonitrile) in turn to remove impurities that may interfere with treatment.	1 mL of 1.5% NaOH solution with constant stirring.	3 mg of fabric samples	1 mL	80 °C	1 h After 1 h, the sample was cool down, neutralized by adding 300 µL of 1 M HCl solution, and filtered with a polytetrafluoroethylene (PTFE) syringe filter. Next, the samples were analyzed using HPLC-HRMS	[39]
		Enzymatic Digestion Method Before treatment, the degraded fabrics were washed by 1 mL of water, 1 mL of methanol, and 1 mL of acetonitrile. Finally, the washed fabric samples were dried at RT and were ready for enzymatic treatment.	100 μL of 3 M NaOH solution was added to the vial, and the vial was placed in a container containing ice for 4 h. Then, the NaOH solution was removed, and 500 μL of 0.5 M acetic acid was added and incubated for 1 min; then, acetic acid was removed and 1.5 mL of buffer solution (0.1 M sodium acetate, pH 5 with acetic acid) was added and kept for 1 min; the buffer solution was removed and 1 mL of enzyme solution (90-g cellulase in 50-mL buffer) was added. The vials were sealed and placed in a shaking bath	3 mg of fabric samples	1 mL	50 °C	24 h After 24 h, the vials were removed from the shaker and sonicated for 30 s, the solutions were filtered with a polytetrafluoroethylene (PTFE) syringe filter. Next, the samples were analyzed using HPLC-HRMS	

n.d.—not determined/not presented.

**Table 3:** Overview of chromatographic methods described in the literature for the identification of reactive dyes extracted from cotton fibers (chromatographic conditions).

No. Method	Analytes	Technique	Column	The Mobile Phase	Gradient Program	The Mobile Phase Flow Rate	Injection	Lit.
**1**	Table 2 No. 5	UPLC	BEH C18 column (1.7 μm, 2.1 × 50 mm, Waters Acquity UPLC^®^) heated to 40 °C	A—10 mM ammonium acetate (pH 9.3) B—acetonitrile	UPLC-DAD-MS/MS 0 min 95% A; 5% B 0–2 min 50% A; 50% B 2–3 min 95% A; 5% B 3–5 min 95% A; 5% B	0.4 mL/min	10 μL	[14]
**2**	Table 2 No. 6	HPLC-DAD-HRMS	a Zorbax Eclipse Plus C18 (2.1 × 50 mm, 3.5 μm) column Pre-column a Zorbax Eclipse Plus C18 narrow bore guard column (2.1 × 12.5 mm, 5 μm) temp. 40 °C	A—20 mmol/L ammonium formate with formic acid in water (pH = 4) B—70/30 MeOH/ACN	3% B from 0 to 1 min, 3%–60% B from 1 to 1.5 min, 60%–90% B from 1.5 to 7 min, holding 90% B from 7 to 9 min then returning to 3% B at 9 to 9.5 min. A 4 min post-run of 3% B was used to re-equilibrate the column	0.5 mL/min	10 μL	[35]
**3**	Table 2 No. 7	HPLC	Grom-sil 120 ODS-5 ST (3 μm, 2 × 150 mm) Grace Davison Discovery Sciences, Deerfield, USA Precolumn AJO-4286 Temp. 22 °C	A—10 mM ammonium acetate in water: MeOH (95:5, *v*/*v*) B—25 mM ammonium acetate in ACN: MeOH (50: 50, *v*/*v*)	50% B (0–53 min) 100% B (53–67 min)	n. d.	10 μL	[36]
**4**	Table 2 No. 8	HPLC	Grom-sil 120 ODS-5 ST (150 × 2.0 mm i.d., 3 μm) Grace Davison Discovery Sciences, Deerfield, USA Precolumn AJO-4286 Temp. 22 °C	A—10 mM ammonium acetate in water: MeOH (95:5, *v*/*v*) B—25 mM ammonium acetate in ACN: MeOH (50:50, *v*/*v*)	Linear gradient Total time 78 min	n. d.	20 μL	[37]
**5**	Table 2 No. 10	HPLC	Zorbax Eclipse Plus C18 (2.1 × 50 mm, 3.5 μm) column with a Zorbax Eclipse Plus C18 narrow bore guard column (2.1 × 12.5 mm, 5 μm); Temp. 40 °C	A—water with 20-mM ammonium formate and formic acid (pH = 4) B—70:30, *v*/*v*) methanol/acetonitrile	3% B from 0 to 1 min, 3%–60% B from 1 to 1.5 min, 60%–90% B from 1.5 to 7 min, holding at 90% B from 7 to 9 min, and 3% B at 9 to 9.5 min. A 4-min post run of 3% B before the next run was performed.	0.5 mL/min	10 μL	[39]
**6**	Cibacron Yellow F-4G Cibacron Blue F-R	Ion-pair HPLC	Nucleosil 100–5, C18, 150 × 4.6 mm I.D. analytical column Macherey-Nagel GmbH & Co.KG	A 47:53 *v/v* mixture of acetonitile and 0.05 M ammonium acetate buffer containing 1 mM trimethylammonium bromide (CTAB) ion-pairing agent	isocratic mode	0.8 mL/min each dye eluted separately 0.6 mL/min a mixture of dyes	n.d.	[43]
**7**	The C.I. Reactive Red 195, C.I. Reactive Yellow 145, C.I. Reactive Blue 221	HPLC	BDS Hypersil C18 column (150 mm × 4.6 mm, 5 µm)	A—90% deionized water and 10% acetonitrile containing 0.1% ammonium acetate (of buffer of pH 6). B—90% acetonitrile and 10% deionized water containing 0.1% ammonium acetate of buffer of pH 6	0.00 min 100% A; 0% B 10.00 min 0% A; 100% B 10.10 min10 0% A; 0% B 20.00 min 0% A; 0% B	0.75 mL/min	20 μL	[44]
**8**	The C.I. Reactive Red 195, C.I. Reactive Yellow 145, C.I. Reactive Blue 221	HPLC	BDS Hypersil C18 column (150 mm × 4.6 mm, 5 µm)	A—90% deionized water and 10% acetonitrile containing 0.1% ammonium acetate (of buffer of pH 6). B—90% acetonitrile and 10% deionized water containing 0.1% ammonium acetate of buffer of pH 6	0.00 min 100% A; 0% B 10.00 min 0% A; 100% B 10.10 min10 0% A; 0% B 20.00 min 0% A; 0% B	0.75 mL/min	20 μL	[45]
**9**	Reactive orange 16 (RO16)	HPLC-MS/MS	Symmetry C18 (50 mm × 1.0 mm I.D., 3.5 μm, Ireland)	(A) 0.1% Acetic acid in water (B) 0.1% Acetic acid in acetonitrile	From 95:5 A:B (*v*/*v*) to 24:76 A:B (*v*/*v*)A (0–15 min), 100% B (for 0.1 min and retention for 2 min), 95:5 A:B (*v*/*v*) (for 0.1 min and retention for 2 min)	0.3 mL/min	10 μL	[46]

n.d.—not determined/not presented.

**Table 4:** Overview of chromatographic methods described in the literature for the identification of reactive dyes extracted from cotton fibers (selected qualification and quantification parameters).

No.	Analytes	Technique	Detector	Scanned Wavelength Range	Retention Time	Value [*m*/*z*]	LOD	Lit.
**1**	Table 2 No. 5	UPLC	DAD MS(ESI) tandem quadrupole mass spectrometer MS/MS—Selected Reaction Monitoring (SRM) mode	300–700 nm for Reactive Yellow 160, Reactive Blue 220, and Reactive Orange 72 at 405 nm, 610 nm, and 478 nm, respectively	Several peaks between 0.75 and 2.75 min within which multiple additional components are present	*m/z* 652, 572, 554, and 614 from the analysis the Reactive Yellow 160; Reactive Blue 220 did not did not appear in UPLC-MS; *m/z* 572, 474, 492, and 417 from the analysis of Reactive Orange 72	DAD—0.33–1.42 ppb MS/MS—3 pg to 83 pg 1 mm extract of Reactive Yellow 160 was detected and arguably quantifiable	[14]
**2**	Table 2 No. 6	HPLC	DAD ESI(-)-(Q-TOF)MS	200–800 nm. The main wavelengths for absorbance analysis 254 nm, 620 nm, and 660 nm; the quantitative analysis at 620 nm	4.6 min RB19-OH Degraded products were also monitored	Q-TOF MS RB19-OH a theoretical *m/z* 501.0432; monitored *m/z* 501.0432	0.12 ± 0.07 µg/mL (DAD) n.d. (HRMS)	[35]
**3**	Table 2 No. 7	HPLC	Diode array detection (DAD) MS(ESI)-LTQ Orbitrap (HRMS)	200–800 nm λ_max_ 494 nm for Reactive Orange 16 λ_max_ 417 nm for Reactive Yellow 145 λ_max_ 540 nm for Reactive Red 120 150–2000 *m/z*	Between 14.5 and 24.8 min	Calculated mass [*m/z*] e.g., Reactive Orange 16 pure *m/z* 572.00980 *m/z* 474.04242 Reactive Orange 16 fiber *m/z* 774.14806 *m/z* 816.15863 (the dye molecule connected to two cellulose units)	e.g., Reactive Orange 16 DAD—37.3 µg/L powder/0.06 mm fiber HRMS—4.2 µg/L powder/0.011 mm fiber	[36]
**4**	Table 2 No. 8	HPLC	DAD MS(ESI)-LTQ Orbitrap (HRMS)	200–800 nm 150–2000 *m/z*	Reactive Orange 122 16.4 min 17.5 min 19.9 min Reactive Orange 195 16 min 14.2 min 17.5 min	Reactive Orange 122 584.5629 [M+2glu-2H]^2-^ 746.616 [M+4glu-2H]^2-^ -(red dye) 624.5407 [M+2glu-2H]^2-^ -(yellow dye) -(blue dye)	n.d.	[37]
**5**	Table 2 No. 10	HPLC	Diode array detection (DAD) MS(negative ESI)-QTOF (HRMS)	200 to 800 nm. The main wavelengths for absorbance analyses were set to 254, 515, 610, 620, and 660 nm 200–800 nm (λ_max_ 515 nm for RR198 λ_max_ 620 nm for RBlk5 λ_max_ 600 nm for RB49 λ_max_ 430 nm and 254 nm for RO35 100–1600 *m/z*	RR198 45.6 min 51.0 min	The hydrolyzed form of dye RR198 at *m/z* 397.5020 (doubly charged deprotonated ion) The hydrolyzed form of dye RBlk5 at *m/z* 370.5093 (doubly charged deprotonated ion) The hydrolyzed form of dye RB49 at *m/z* 397.5354 (doubly charged deprotonated ion) The hydrolyzed form of dye RO35 at *m/z* 363.5312 (doubly charged deprotonated ion)	n.d.	[39]
**6**	Cibacron Yellow F-4G Cibacron Blue F-R	Ion-pair HPLC	UV absorbance	275 nm	Blue 4.70 min Hydrolyzed Blue 3.44 min Yellow 3.32 min Hydrolyzed Yellow 2.38 min Blue in mixture (m) 7.54 min Hydrolyzed Blue (m) 5.07 min Yellow in mixture (m) 4.57 min Hydrolyzed Yellow (m) 3.16 min		n.d.	[43]
**7**	The C.I. Reactive Red 195, C.I. Reactive Yellow 145, C.I. Reactive Blue 221	HPLC	DAD	200–800 nm λ_max_ 540 nm Reactive Red 195 λ_max_ 420 nm Reactive Yellow 145 λ_max_ 610 nm Reactive Blue 221	Total runtime of 20 min e.g., several peaks with different RT belonging to different dye forms for Disperse Red 195 t_R_ = 1.77 min; t_R_ = 2.75 min; major peak at t_R_ = 5.54 min attributed to the active form of Reactive Red 195. In the opposite, the Reactive Yellow 145 and Reactive Blue 221 show only one big peak respectively at RT 6.71 min and RT 6.64 min		n.d.	[44]
**8**	The C.I. Reactive Red 195, C.I. Reactive Yellow 145, C.I. Reactive Blue 221	HPLC	DAD	200–800 nm λ_max_ 540 nm Reactive Red 195 λ_max_ 420 nm Reactive Yellow 145 λ_max_ 610 nm Reactive Blue 221	Total runtime of 20 min e.g., several peaks with different RT belonging to different dye forms for Disperse Red 195 t_R_ = 1.79 min, 2.2 min, 2.34 min, 2.92 min, 3.55 min and 4.11 min—the inactive forms; two major peaks at t_R_ = 4.73 min and t_R_ = 5.96 min attributed to the partially hydrolyzed forms of Reactive Red 195. Peak at t_R_ = 5.33 min attributed to the completely hydrolyzed form. In the opposite, the Reactive Yellow 145 and Reactive Blue 221 showed only one big peak respectively at RT 2.74 min and RT 2.71 min of the inactivated forms and at 5.15 min (RY145), 5.24 min, 5.91 min (RB221) of the partially hydrolyzed forms and at t_R_ = 6.30 min attributed to the completely hydrolyzed form of RB221		n.d.	[45]
**9**	Reactive orange 16 (RO16)	HPLC-MS/MS	MS(ESI) quadrupled type tandem MS MS/MS–MRM mode	n.d.	5.09 min	Molecular weight 617.54 285.4 [M-2H]^2-^ 285.4→236.85 285.4→264	MS/MS—2.10 ng/mL	[46]

n.d.—not determined/not presented.
